# Evaluation of Successfulness of Capacity Building Programmes on Smokeless Tobacco and Areca Nut Cessation 

**DOI:** 10.31557/APJCP.2021.22.4.1287

**Published:** 2021-04

**Authors:** Ruwan Duminda Jayasinghe, P.R. Jayasooriya, Hemantha Amarasinghe, PVKS Hettiarachchi, BSMS Siriwardena, UKYGDM Wijerathne, SK Kithalawarachchi, WM Tilakaratne

**Affiliations:** 1 *Department of Oral Medicine and Periodontology, Faculty of Dental sciences, University of Peradeniya, Sri Lanka. *; 2 *Centre for Research in Oral Cancer, Faculty of Dental Sciences, University of Peradeniya, Sri Lanka. *; 3 *Department of Oral Pathology, Faculty of Dental Sciences, University of Peradeniya, Sri Lanka. *; 4 *Oral Health Unit, Family Health Bureau, Colombo 10, Sri Lanka. *; 5 *Oral and Maxilliofacial Clinical Sciences, Faculty of Dentistry, University of Malaya, Malaysia. *

**Keywords:** Tobacco, smokeless tobacco, Areca nut, cessation, dental surgeons

## Abstract

**Background:**

Prevalence of smoking in Sri Lanka has shown a gradual reduction whilst the use of smokeless tobacco and areca nut exhibits an increasing trend. At present, only a few well-structured smokeless tobacco (SLT)/areca nut (AN) cessation programs have been conducted in Sri Lanka, which is a gross underachievement as betel chewing-related oral squamous cell carcinoma is the most common cancer in Sri Lankan males. As General Dental Practitioners (GDP) do not contribute significantly to SLT/AN cessation activities at present, capacity building programs on SLT/AN control were carried out. The study evaluated the knowledge, attitude and practices imparted on SLT/AN control among dental surgeons.

**Methods:**

Following a single day capacity building program on smokeless tobacco / areca nut control, two self-administered questionnaires were used to assess the improvement of knowledge and change of attitudes among 663 GDPs.

**Results:**

Majority had a good knowledge on harmful effects of SLT but not on areca nut. Knowledge of the current legislation on SLT control in Sri Lanka and carcinogenicity of areca nut was not satisfactory. Almost all agreed that proper counseling leads to patient quitting the habit, a formal training is necessary to conduct tobacco control activities and it should be a part of the regular treatment modalities. More than 80% of the participants support strict legislation. Most important factors leading to poor involvement in tobacco cessation activities were lack of expertise and inadequate educational material and not breach of patient privacy and lack of financial incentives. 20.1% dental surgeons had consumed smokeless tobacco / areca nut products in the past and only a few were current users of tobacco and/or areca nut.

**Conclusions:**

Well planned workshops are efficient in improving knowledge, practices and attitudes of dental surgeons towards SLT/AN cessation.

## Introduction

Sri Lankans use betel quid containing betel leaves, areca nut, slaked lime (aqueous calcium hydroxide paste) and dried tobacco leaves. Other than the traditional betel quid, commercially available SLT products such as pan masala, mawa, red tooth powder, khaini, tobacco powder, zarda and many others have shown significant increase in the recent years especially among adolescents (Somatunga et al., 2012). Betel chewing practice is detrimental to health as its use has been directly linked with the development of oral potentially malignant disorders (OPMDs) and oral cancer. However, it is difficult to combat betel chewing practice in Sri Lanka as it is culturally ingrained in the Sri Lankan society. In addition, most people are unaware of the harmful effects of areca nut which is a group I carcinogen and continues to use it due to social values and beliefs (Amarasinghe et al., 2010; Amarasinghe et., 2010; Sotto et al., 2020). 

In a survey conducted in 2012, 15.8% of Sri Lankans which includes 8.6% of the youth were found to be smokeless tobacco (SLT) users (Somatunga et al., 2012). To reduce SLT/AN use in Sri Lanka, the project Committee of the National Authority of Tobacco and Alcohol (NATA), issued a gazette notification banning the production, distribution and sale of SLT products in the year 2016. The gazette notification has not been successful in reducing the SLT/AN use to the expected levels. It is therefore important to explore other avenues to combat the menace of SLT/AN use.

Further, according to the 2018 WHO factsheet on Sri Lanka (www.who.int/news-room/fact-sheets/detail/tobacco), tobacco is responsible for 12,351 deaths and it represents 10% of all deaths. It has estimated that there are 2.1 million current tobacco users in the country.

Use of smokeless tobacco as part of betel quid is a widely practiced risk habit in Sri Lanka. Multiple initiatives such as legislation prohibiting public smoking, increased taxation on tobacco products taken by the successive governments together with the efforts of health care workers and some non-governmental organizations have resulted in a gradual reduction in the number of smokers in the country. Smoking prevalence among males in Sri Lanka was 39% in 2009 which reduced to 28.4% in 2015 (www.searo.who.int/tobacco/documents).

In contrast to smoking, SLT use is gaining popularity, especially among the youth. In addition, the use of areca nut with the traditional betel quid has become a popular habit. Areca nut has been identified as a group I carcinogen by IARC. In most instances, it has been used as a part of the betel quid but there’s a trend especially among youth to use commercially prepared areca nut packets (Karunarathne & Ekanayake, 2016). The harmful effects of SLT use have been well documented and include a high prevalence of OPMD (Oral potentially malignant disorders) and oral squamous cell carcinoma (OSCC). It is the number one ranked cancer with high mortality out of all cancers among Sri Lankan males (National Cancer Control Programme Sri Lanka. Cancer Incidence Data: Sri Lanka Year 2009, NCCP 2009). Public awareness on oral cancer and OPMD as well the risk habits contributing to those lesions appear to be not satisfactory (Amarasinghe et al., 2010; Amarasinghe et al., 2010). 

There had been several attempts to develop a successful SLT cessation model (Moss et al., 2015). However, it is unfortunate that successful SLT cessation programs that would suit the Sri Lankan population are yet to be developed. From the patients’ point of view, once they are offered SLT cessation advice, lack of time, lack of rapport with the individuals who conduct SLT/AN control programs, and accessibility to cessation centers have been identified as significant shortcomings (Sotto et al., 2020). 

According to the management protocols of OPMD established in Sri Lanka (Guidelines for management of oral potentially malignant disorders. National cancer control program, Sri Lanka, 2019), low-risk OPMDs are managed at the primary care level by GDPs with education on habit intervention with 6-month review appointments. Therefore, tobacco and areca nut cessation advice has to be considered as an important duty of a dental surgeon. Dental surgeons are well suited and can play a major role in tobacco cessation activity and the dental clinic is an ideal place to implement it (Albert et al., 2002; Saddichha et al., 2010; Chandrashekar et al., 2011; Mohanty et al., 2013). Lack of knowledge and experience has been identified as an important barrier in carrying out tobacco cessation activities by dental surgeons (Binnal et al., 2012).

With these shortcomings in mind, island-wide capacity-building workshops to train healthcare workers on tobacco and areca nut cessation activities were planned and conducted by the Centre for Research in Oral Cancer, Faculty of Dental Sciences, the University of Peradeniya in collaboration with the Presidential Task Force for Drug Prevention and Ministry of Health, Sri Lanka. The present study targeted the participants of these programmes to evaluate the successfulness of the programme concerning a change in their knowledge, attitudes, and practices towards tobacco and areca nut cessation. In addition to the assessment of the application of imparted knowledge and attitudes towards conducting successful SLT/ areca nut cessation programmes in their dental clinics. 

Majority of tobacco cessation protocols have been developed for smoking cessation (Warnakulasuriya, 2002; Carson et al., 2012). However, though the same protocols can be applied for SLT cessation as well, a successful implementation may require some modifications. SLT is used together with areca nut in betel quid, especially in South and South East Asian countries. Areca nut is a known addictive substance. Therefore, tobacco cessation should be targeted together with areca nut cessation. Dependency and withdrawal symptoms of SLT use as well as areca nut use show differences when compared with smoking (Benegal et al., 2008; Mirza et al., 2011). Thus, a training programme on SLT and areca nut cessation was developed to improve the knowledge and skills of dental surgeons targeting the situation and needs of the country. 

The impact of the capacity building workshop was evaluated by the knowledge that the participants gained and/or a change of attitude towards tobacco cessation using a pre-tested questionnaire. The findings will be useful to improve the quality of the training programs to be developed in the future. 

## Materials and Methods

This study was conducted with the general objective of evaluating the improvement of knowledge, impact on the change of attitude related to smokeless tobacco and areca nut cessation among dental surgeons following a one day educational workshop. 

A pre and post-test study were conducted among the Dental surgeons included from all the provinces in Sri Lanka, who participated in the workshop conducted to train them on SLT and areca nut cessation practices. Two self-administered questionnaires were developed to assess dental surgeons’ knowledge and attitudes towards tobacco cessation practices. The first questionnaire was administered prior to the workshop and consisted of five components including, 1. demographic profile of the participants: age, gender, number of years in service and designation, 2. knowledge related to effects of SLT and areca nut with tobacco cessation practices, 3. attitudes regarding smokeless tobacco cessation counseling (TCC) and ways to reduce SLT use, 4. tobacco control measures undertaken by dental surgeons and 5. barriers to implement tobacco cessation counseling in dental clinics (Refer annexure 1a). 

Workshop objectives and agenda of the workshop were developed with a consultative meeting with the experts in the CROC, FDS (Annexure 2). For each lecture-discussion, lesson plans were prepared and the most suitable experts were given the responsibility of conducting the lecture-discussion. Powerpoint presentations were developed by the assigned experts and consensus was obtained from the panel of experts. A tobacco cessation manual was developed and printed by the principal author and distributed among all dental surgeons who participated in the workshop ( Annexure 3). 

These workshops were organized as part of the initiatives initiated by the Centre for Research in Oral Cancer together with the Ministry of Health and Presidential Task Force on Drug Prevention to educate and train health care workers on SLT and areca nut cessation. These workshops were conducted as a half a day workshop in all provinces of the country with more than one program for larger provinces by experienced and well-trained specialists in the fields of Oral Medicine, Oral Pathology and Community Dentistry. When conducting the said workshops, prior arrangements were made on the dates convenient for the regional dental surgeon (RDS). All the workshops were conducted by the same group of subject experts and was composed of lectures on SLT/AN related disease burden of the country, health effects of SLT/AN, legislation on tobacco and areca nut in Sri Lanka, lectures were followed by a role paly on tobacco cessation methods with group work and open discussion. Self – administered questionnaires were developed in English language and the content validations for these questionnaires were carried out with the help of three consultants in Community Dentistry. Pre-testing of questionnaires was conducted among ten dental surgeons working in the University Dental Hospital Peradeniya for each questionnaire and these responses were excluded from the main study. All the dental surgeons who were participating in these training workshops were recruited for the study after obtaining their informed consent. An information sheet containing measures to reduce the non-compliance and possible Hawthorne effect: confidentiality was assured for the group and identity was not requested (Annexure 4).

The second questionnaire was given immediately after the completion of the workshop which contained the same questions on knowledge and impact on and attitude component of the pre-workshop questionnaire with another component specifically targeting the training program. This section included questions on the workshop and open-ended questions to identify the most valuable and least valuable components of the workshop and suggestions for improvement of the workshop (Refer Annexure 1b). Dental surgeons who submitted an incomplete questionnaire with less than 70% responses were excluded from the study.

*Data analysis*

The SPSS (version 22) software package was used for the data analysis. Data obtained from the questionnaire were entered in SPSS software and were expressed as frequencies (percentages) using descriptive statistics. The improvement of the knowledge following the workshop was assessed by comparing the pre vs. post workshop responses using McNemar test. Attitudinal questions were weighted and amalgamated to produce total attitudinal value for each subjects. Total values were converted to Z scores and dichotomized to positive and negative attitude. Pre and post dichotomized values were tested for significance by McNemar test. P-values of less than 0.05 were considered statistically significant.

Ethical clearance for the study was obtained from the Ethics Review Committee of the Faculty of Dental Sciences, University of Peradeniya.

## Results

A total of 663 questionnaires were considered in the analysis. This study group had a female preponderance with the age ranges from 28 to 60 years with the majority belonging to above 35 years. Out of the participants, 490 (73.9%) had never consumed tobacco in any form in their lifetime and 133 (20.1%) had a past habit of tobacco consumption.

Among the participants, 14 (2.1%) and 15 (2.3%) had consumed only SLT and areca nut respectively. The majority (2.7%) of the participants had initiated the habit between the ages of 16 – 20 years and practiced the habit for 1-5 years (22%). The most common reasons for tobacco consumption were recorded as part of the culture and to keep the company with friends. Only 180 (27.1%) dental surgeons had received formal training in tobacco cessation methods.

Analysis of the knowledge related to Smokeless tobacco, its effects, and cessation practices There were ten questions to assess the knowledge of dental surgeons and, eight questions demonstrated a statistically significant increase in knowledge (P<0.05) following the workshop when compared to the pre-workshop knowledge., None of the questions had at least 80% correct response and six questions had less than 50% correct responses prior to the workshop. Therefore, the majority didn’t know the facts, that “Red tooth powder is an SLT popular among children” (16.1%), “Manufacturing and selling of SLT products is banned in Sri Lanka” (48%), “Four people die each day in Sri Lanka due to SLT use” (36.8%), “Withdrawal symptoms may prevent SLT users from quitting the habit” (42.5%), “SLT users can be given existing drugs prescribed to combat cigarette craving” (21.3%), and “Five “R” concept can be used in counseling a client who is unwilling to quit SLT use” (33.2%). Accordingly, a significant increase of knowledge was observed for all ten questions, following the workshop (P<0.001) (Table 1).

*Attitudes regarding smokeless tobacco cessation counseling (TCC) and ways to reduce SLT use*

According to the responses of the questions on the Likert scale, the total weighted score was calculated. Pre-workshop total attitudinal score: a range of 9 (16- 25) with the mean value of 22.15 (SD 2.3) and post-workshop: a range of 6 (14-20) with the mean of 18.7 (SD 1.56). These total scores were converted to Z scores. The total Z scores were dichotomized: Less than zero as ‘unsatisfactory attitudes’ and above zero as ‘satisfactory’ attitude. McNemar test was carried to test the significance of the differences of pre and post-workshop assessment. Table 2 shows the Pre and post dichotomized attitude scores. 

With these two correlated samples, the change of attitude in pre and post assessment was highly significant (P<0.001).

*Tobacco control measures undertaken by Dental surgeons*

There were six different questions to assess this section and only 283 (42.7%) had the habit of routinely inquiring regarding tobacco use from all patients. Almost 50% enforced a tobacco product free environment in their clinics. 233 (35.1%) dental surgeons provide advice against SLT use when they identify a patient to be a SLT user even without any lesion present in the oral cavity due to SLT use. In contrast, 5.7% of the dental surgeons only provided any advice if the patient had a lesion due to SLT use. Further, 34.2% of the dental surgeons practice tobacco cessation counselling in their clinics using patient education via audio and video materials, providing leaflets to all patients to read followed by discussing with them the consequences and motivation to cease SLT/AN use in detail. 

Only 127 (19.2%) claim that they were unsuccessful in their tobacco cessation counselling. 

*Barriers encountered to implement tobacco cessation counseling*

Out of the six reasons given, the two most common reasons were lack of educational materials (39.7%) and lack of expertise (30.8%). (Table 3)

**Table 1:** Knowledge of Dental Surgeons before and after the Workshop

Knowledge Question	Correct answers Pre workshop No (%)	Correct answers Post workshop No (%)	P value (McNemar test)
SLT use is increasing in popularity among youth and adolescents.	526 (79.3)	641 (96.7)	<0.001
Areca nut is an addictive substance.	460 (69.4)	630 (95)	<0.001
Red tooth powder is a SLT popular among children.	107 (16.1)	388 (58.5)	<0.001
Manufacturing and selling of SLT products is banned in Sri Lanka	318 (48.0)	542 (81.7)	<0.001
Areca nut is a group-I carcinogen in humans.	348 (52.5)	556 (83.9)	<0.001
Four persons die each day in Sri Lanka due to SLT use.	244 (36.8)	401 (60.5)	<0.001
Withdrawal symptoms may prevent SLT users from quitting the habit.	282 (42.5)	503 (75.9)	<0.001
SLT users can be given existing drugs prescribed to combat cigarette craving	141 (21.3)	394 (59.4)	<0.001
Five "R" concept can be used in counseling a client who is unwilling to quit SLT use	220 (33.2)	493 (74.4)	<0.001
SLT use in pregnancy may result in low birth weight babies and still births	387 (58.4)	506 (76.3)	<0.001

**Table 2.** Change of Attitude between Pre and Post Workshop

Pre test	Post test			P value (McNemar test)
	Satisfactory attitudes N (%)	Unsatisfactory attitude N (%)	Total N (%)	
Satisfactory attitudes N (%)	120 (29.3)	23 (5.6)	143 (35)	0.001
Unsatisfactory attitude N (%)	151 (36.9)	115(28.1)	266 (65)	
Total N (%)	271(66.3)	138 (33.7)	463 (100)	

**Table 3:** Identifed Barriers by the Dental Surgeons

Barrier	Number	%
I don’t have enough time	181	27.3
Lack of expertise	204	30.8
Tobacco cessation is low priority to me	52	7.8
Respect of patient privacy	151	22.8
Lack of educational material	263	39.7
Lack of financial reimbursement	123	18.6

## Discussion

Tobacco cessation counseling has been identified as an effective method of promoting the users to give up the habit and it is considered as part of the routine work of health care professionals. There are multiple interventions for tobacco cessation. There had been extensive work on smoking cessation but research on smokeless tobacco cessation is scarce (Nethan et al., 2018). Most of the interventions appear to be effective against both smoking and smokeless tobacco use. Behavioural interventions are highly efficient in SLT cessation but regular telephone support/quit-lines also appeared to be beneficial in addition to some pharmacological interventions (Nethan et al., 2018).

There are multiple action plans in practice to minimize the use of tobacco in Sri Lanka, but most of these initiatives are targeted at smoking. With the initiatives of the Smokeless Tobacco Project Committee of the National Authority of Tobacco and Alcohol (NATA), a gazette notification has been issued banning the production, distribution, and sale of SLT products in the year 2016. However, this important legislation has not being implemented effectively in the country. Awareness among both public and health care professionals appears to be inadequate and supporting this fact 48% of the dental surgeons who participated in this survey were not aware of this regulation.

To the best of our knowledge, this study is the first of this nature to identify knowledge, attitudes and practices regarding TCC among dental surgeons in Sri Lanka. We will be using the findings of this study to improve tobacco cessation counseling in the country by introducing appropriate programs. Our sample size was 663 which is more than 25% of the dental surgeon strength in Sri Lanka. Therefore, the findings of this study can be considered as a representation of the situation in the country. 

As SLT usage is common in Sri Lanka and also on the rise, it’s important to promote SLT cessation. Dental surgeons are identified as an ideal group of health professionals to carry out SLT cessation activities. Brief intervention at the dental office is very effective in preventing SLT use among the general population (Victor et al., 1995). Brief interventions or behavioral change interventions are useful in both smoking and smokeless tobacco cessation (Nethan et al., 2018; Siddiqi et al, 2016). Formal training is mandatory to initiate tobacco cessation. Binnal et al., in their study conducted in India with house surgeons in one dental institute have reported that 97% of the participants in their study were willing to undertake tobacco cessation activities but 93% of them were lacking adequate training (Binnal et al., 2012). We have observed that only 27.1% of the dental surgeons in this study have received any formal training in tobacco cessation highlighting the importance of organizing more and more such programs for dental surgeons and other health care professionals. Findings of the pre-workshop questionnaire evaluation demonstrated that a significant percentage of dental surgeons are not having sufficient knowledge especially on some aspects of SLT cessation. Analysis of post-workshop questionnaire showed that the dental surgeons improved their knowledge significantly demonstrating the effectiveness of the workshops conducted. However, as the assessment was done immediately after the workshop, this will not indicate a long-term effectiveness. A separate study is being planned to identify the long-term success of the workshop.

Dental surgeons are in an ideal position to advise their patients regarding the ill effects of tobacco and it must be considered as part of their routine work. Having proper attitudes towards tobacco cessation is an essential requirement for the successful implementation of such programs, similar to many other studies conducted around the world (Binnal et al., 2012; Bangera et al., 2018; Al-Maweri et al., 2018). Sri Lankan dental surgeons had a satisfactory level of good attitudes towards tobacco cessation. This aspect has shown a statistically significant improvement after the workshop highlighting that properly planned workshops can be used to improve not only knowledge but also attitudes of dental surgeons towards tobacco cessation. We observed that only 42.7% of the respondents had the habit of inquiring regarding tobacco usage from all of their patients routinely and this is comparatively less than the 83.5% reported by Al-Maweri et al., (2018). Giving up the SLT habit early will definitely benefit the patient as the chances of developing adverse effects increase with the duration of SLT use. In this regard, it is encouraging to observe that 35.1% of the dental surgeons provide advice against SLT use for SLT users in their clinics even when they are without any oral mucosal lesions. However, there is room for further improvement as compared to literature this good practice is less often implemented by Sri Lankan dental surgeons (Al-Maweri et al., 2018). Furthermore, 5.7% of the respondents provide tobacco cessation advice only when the patient is having a lesion due to SLT use, which is unacceptable as each person with SLT/AN use should receive TCC.

Maintain a tobacco-free environment in the health care institution is important to motivate the patients to give up the habit. The Ministry of Health as well as the Ministry of Public Administration of Sri Lanka has issued relevant circulars banning the use of tobacco in government institutions in Sri Lanka. Dental surgeons must play a leading role in implementing these circulars in their institutions. According to our findings, only 50% of the participants enforce a tobacco product-free environment in their clinics. 

There are multiple methods of providing knowledge on tobacco cessation to the patient. Behavioural interventions by health care professionals alone have shown high efficacy in SLT cessation and it has been identified as the most suitable tobacco cessation intervention for countries with low-resources but high SLT burden (Nethan et al., 2018). Information on SLT cessation interventions is minimal and therefore, research in this area must be encouraged to identify the most appropriate intervention. Al-Maweri et al., (2018) reported that around half of the Yemeni dental surgeons in their study believed that tobacco cessation intervention may affect their clinical practice and reduce their income and they also believed that provision of dental treatment is more important than providing TCC. Similar findings were reported by others as well (Parakh et al., 2013). In contrast, only a small minority of Sri Lankan dental surgeons in the present study thought that it can affect their income and clinical practice whereas the majority believed that providing TCC is equally important as any routine dental treatment.

In addition to the tobacco cessation counseling by a trained dental surgeon, extra educational material is very helpful. Extra educational materials such as leaflets, booklets, posters audio, video materials are useful in providing tobacco cessation message to the users. It can provide the message more simply and understandably and patients can read/ listen to them while at rest or at their convenience. Some of the dental surgeons who participated in this study practice tobacco cessation counselling in their clinics together with patient education using audio, video materials and by giving leaflets to all patients. Around 40% of the respondents have identified a lack of educational materials as the main barrier in implementing tobacco cessation counseling. Therefore, this fact has to be taken seriously by the relevant authorities and necessary steps have to be taken to develop educational materials for the patients in their languages.

Most dental surgeons have tried to carry out tobacco cessation counselling. However, 19.2% of them claim that they were unsuccessful in their efforts. Lack of experience was considered as an important barrier by 30.8% of the participants in this study highlighting the importance of having regular formal training on tobacco cessation.

Self-use of tobacco by health care professionals has been identified as the main barrier in controlling tobacco use among people. It has a direct impact on the attitude of the dental surgeon towards TCC where non-users were showing better attitudes (Al-Maweri et al., 2018). Most dental surgeons believe that they must refrain from using tobacco products and must act as role models (Parkh et al., 2013). Past users of tobacco in the present study were as high as 15.2% with 2.1% had used SLT. There are only a very few dental surgeons who are current tobacco and/ or areca nut users. This is completely different from most of the studies reported where a significant percentage of dental surgeons use tobacco, especially smoking (Al-Maweri et al., 2018). 

*Limitations*

There were some limitations of the study. We have conducted capacity-building workshops targeting all the dental surgeons in the area but some were not present in them due to the service requirements. In most instances experienced dental surgeon has to stay back in the hospital to provide the services to the patients hence there can be a selection bias of the sample. A questionnaire that we used for the study was pre-tested but was not a validated one. Another limitation of the study includes some incompletely answered questions in questionnaires which may lead to non-response bias. In addition, the effectiveness of the workshops was only assessd immediately after the workshop and therefore long term effectiveness was not assessed. Similar to any questionnaire-based study, we have collected self-reported data from the participants and therefore the answers to some of the questions might not be accurate especially the attitude and practice component of the questionnaire. In order to minimize this bias, de-identified questionnaires were used.

In conclusion, smokeless tobacco cessation intervention in the dental clinic is a simple method of promoting people to give up the risk habit. Tobacco cessation counseling should be part of routine dental care. Capacity building workshop is an effective method of training dental surgeons on tobacco cessation and the present study highlights the importance of having proper training programs for dental surgeons on tobacco cessation with special attention on smokeless tobacco and areca nut cessation. 

*Abbreviations*

SLT- Smokeless tobacco

AN- Areca nut

TCC- Tobacco cessation counseling

OPMD-Oral potentially malignant disorders

OSCC- and oral squamous cell carcinoma 

WHO- World Health Organization

NATA- National Authority of Tobacco and Alcohol 

GDP= General Dental Practitioner

## Author Contribution Statement

RDJ conducted literature searches and provided summaries of previous research studies, was involved in conceptualization, Project administration, and wrote the first draft of the manuscript. PJ designed the study and wrote the protocol. HA conducted the formal statistical analysis. SS and KH were involved with initial protocol development, data collection, and editing of the first draft. DW involved with data acquisition and curation. SK involved in fund acquisition and data collection. WMT involved with project administration. Apart from each specific role all the authors contributed to and have approved the final manuscript.

## Data Availability

The datasets used and/or analyzed during the current study are available from the corresponding author on reasonable request.
